# Electrocardiographic ST-Segment Depression and Exposure to Traffic‐Related Aerosols in Elderly Subjects with Coronary Artery Disease

**DOI:** 10.1289/ehp.1002372

**Published:** 2010-10-21

**Authors:** Ralph J. Delfino, Daniel L. Gillen, Thomas Tjoa, Norbert Staimer, Andrea Polidori, Mohammad Arhami, Constantinos Sioutas, John Longhurst

**Affiliations:** 1 Department of Epidemiology, School of Medicine and; 2 Department of Statistics, School of Information and Computer Sciences, University of California–Irvine, Irvine, California, USA; 3 Department of Civil and Environmental Engineering, Viterbi School of Engineering, University of Southern California, Los Angeles, California, USA; 4 Department of Civil Engineering, Sharif University of Technology, Tehran, Iran; 5 Susan Samueli Center for Integrative Medicine, and Cardiology Division, Department of Medicine, School of Medicine, University of California–Irvine, Irvine, California, USA

**Keywords:** aerosols, air, coronary artery disease, epidemiology, longitudinal data analysis, myocardial ischemia, outdoor air, size distribution

## Abstract

**Background:**

Air pollutants have not been associated with ambulatory electrocardiographic evidence of ST-segment depression ≥ 1 mm (probable cardiac ischemia). We previously found that markers of primary (combustion-related) organic aerosols and gases were positively associated with circulating biomarkers of inflammation and ambulatory blood pressure in the present cohort panel study of elderly subjects with coronary artery disease.

**Objectives:**

We specifically aimed to evaluate whether exposure markers of primary organic aerosols and ultrafine particles were more strongly associated with ST-segment depression of ≥ 1 mm than were secondary organic aerosols or PM_2.5_ (particulate matter with aerodynamic diameter ≤ 2.5 μm) mass.

**Methods:**

We evaluated relations of air pollutants to ambulatory electrocardiographic evidence of cardiac ischemia over 10 days in 38 subjects without ST depression on baseline electrocardiographs. Exposures were measured outdoors in retirement communities in the Los Angeles basin, including daily size-fractionated particle mass and hourly markers of primary and secondary organic aerosols and gases. Generalized estimating equations were used to estimate odds of hourly ST-segment depression (≥ 1 mm) from hourly air pollution exposures and to estimate relative rates of daily counts of ST-segment depression from daily average exposures, controlling for potential confounders.

**Results:**

We found significant positive associations of hourly ST-segment depression with markers of combustion-related aerosols and gases averaged 1-hr through 3–4 days, but not secondary (photochemically aged) organic aerosols or ozone. The odds ratio per interquartile increase in 2-day average primary organic carbon (5.2 μg/m^3^) was 15.4 (95% confidence interval, 3.5–68.2). Daily counts of ST-segment depression were consistently associated with primary combustion markers and 2-day average quasi-ultrafine particles < 0.25 μm.

**Conclusions:**

Results suggest that exposure to quasi-ultrafine particles and combustion-related pollutants (predominantly from traffic) increase the risk of myocardial ischemia, coherent with our previous findings for systemic inflammation and blood pressure.

Risk of myocardial infarction has been associated with acute exposure to ambient particulate matter (PM) air pollution in most but not all time-series and case-crossover studies recently reviewed (Bhaskaran et al. 2009). All of these studies have used available ambient air pollution data from regulatory monitoring sites, and in all but a few studies (Peters et al. 2001; Zanobetti and Schwartz 2006), only total particle mass measurements were available [PM ≤ 2.5 μm or ≤ 10 μm in aerodynamic diameter (PM_2.5_ and PM_10_, respectively)]. A recent study of aggregate hospital data in multiple cities has found stronger associations of PM_2.5_ with myocardial infarction in cities where PM_2.5_ was higher in tracers of carbonaceous aerosol sources (traffic, industrial plants, and biomass burning) such as organic carbon (OC) and certain metals (Zanobetti et al. 2009a).

Exposure to traffic-related air pollutants may be particularly important in associations reported in the time series studies (Peters et al. 2004). Traffic-related or other exposures from fossil fuel combustion can vary considerably across space and time, and such exposures by individuals may be poorly represented by ambient data (Sioutas et al. 2005). There is experimental evidence supporting the hypothesis that components of vehicular exhaust play a role in inducing cardiac ischemia in subjects with coronary artery disease (Mills et al. 2007). However, spatially resolved data on particle composition, including carbonaceous aerosols, are usually unavailable in epidemiologic studies.

Ultrafine PM (≤ 0.1 μm in diameter) is generally found at higher concentrations near combustion sources such as highways (Sioutas et al. 2005). Data on this particle size are also rarely available. Ultrafine PM may have greater potential to induce oxidative stress and inflammation than larger PM that makes up much of PM_2.5_ mass, because of higher concentrations of redox-active primary organic components (Ntziachristos et al. 2007), higher deposition efficiency in small airways, and magnitudes higher particle number concentration and surface area (Oberdörster et al. 2005). Furthermore, hourly speciated PM exposure data are rarely available but could help us to understand differences in effects of acute hourly versus longer-term PM exposures and to identify the effects of community exposure sources and composition (Lippmann 2009). An understanding of these issues is important to better protect public health because current U.S. federal regulation of PM air pollution is based on 24-hr and annual averages of PM_2.5_ and PM_10_ mass.

We followed a cohort panel of 38 elderly subjects with a history of coronary artery disease using 10 days of continuous ambulatory electrocardiograph (ECG) monitoring. To address the above issues, we collected repeated hourly measurements of markers of traffic-related air pollution as well as daily measurements of size-fractionated particle mass. We evaluated the relation of ST-segment depression of ≥ 1 mm (0.1 mV) to these air pollutant exposures in the outdoor home environment of subjects. ST-segment depression at ≥ 1 mm during ambulatory ECG monitoring and exercise testing likely represents cardiac ischemia and is predictive of cardiac morbidity and mortality among people at high risk (Gibson et al. 2007).

We specifically aimed to evaluate whether exposure markers of primary organic aerosols and ultrafine PM were more strongly associated with ST-segment depression of ≥ 1 mm than were secondary organic aerosols or PM_2.5_ mass. Primary organic aerosol is derived from fossil fuel combustion sources, which in the study area is dominated by traffic. We also used nitrogen oxides [NO_x_; nitric oxide (NO) + nitrogen dioxide (NO_2_)] and carbon monoxide (CO) as markers of primary combustion-related air pollution. Secondary organic aerosol derives from products of photochemical reactions and aerosol aging involving reactions of volatile and semivolatile organics from anthropogenic and biogenic sources. We also used ozone (O_3_) as a marker of high photochemical activity. We tested coherence with our previous analyses showing that, compared with O_3_ and exposure markers of secondary organic aerosols, markers of primary combustion-related gases and organic aerosols were more strongly associated with circulating biomarkers of systemic inflammation (e.g., interleukin-6) (Delfino et al. 2008, 2009, 2010a, 2010b) and with ambulatory systolic and diastolic blood pressures (Delfino et al. 2010c). We also investigated differences in associations of ST-segment depression with recent hourly versus daily to multiday average exposures that could have implications regarding mechanisms of pollutant effects. We anticipated that both very acute and longer term exposure–response relations would be observed based on experimental data (Mills et al. 2007; Törnqvist et al. 2007).

## Materials and Methods

### Population and design

This was a study of within-subject repeated measures in which each subject acted as his or her own control. We recruited subjects from four retirement communities located in the Los Angeles Air Basin. Subjects were eligible if they had a confirmed history of coronary artery disease, were ≥ 65 years old, were a nonsmoker, and had no exposure to environmental tobacco smoke. Study cardiologists with the aid of research nurses clinically evaluated 105 potentially eligible subjects in our mobile medicine clinic. Twenty-one subjects were not eligible and 18 dropped out, leaving 66. Twenty-eight of these subjects were excluded from the present analysis of ambulatory ECG data for ST-segment depression for the following reasons (several subjects had two precluding conditions): digoxin use (*n* = 8); abnormalities on baseline 12-lead resting ECG, including left bundle branch block (*n* = 10); nonspecific ST-T wave abnormalities (*n* = 10); intraventricular conduction delay (*n* = 4, one with diffuse repolarization changes, three with nonspecific ST-T wave abnormalities); left ventricular hypertrophy (*n* = 3); and right bundle branch block (*n* = 2). None had uncontrolled diabetes. Among the remaining 38 subjects included in the present analysis, 18 exhibited ST-segment depression (≥ 1.0 mm). Of 7,273 hr of data available for analysis, we observed 403 hr (5.5%) of ST depression in the ambulatory ECG data.

Two communities were studied in 2005–2006, and two communities were studied in 2006–2007. We followed subjects in two seasonal periods to increase variability in exposures to PM components (including primary and secondary organic aerosols) and in size distribution by season (Sioutas et al. 2005). In each community, we collected data during a period of higher photochemical activity (July to mid-October) and during a cooler period when traffic-related primary air pollutants increase at ground level (mid-October through February). Subjects were studied with ambulatory ECG monitoring in two periods of 5 consecutive days during each of the two seasonal periods. Ambulatory monitoring started Monday morning and ended Friday afternoon. Daily home visits by a research assistant took place for downloads of electronic data, including ambulatory ECG, actigraphs, and personal digital assistant diaries.

The research protocol was approved by the Institutional Review Board of the University of California–Irvine. We obtained informed written consent from subjects.

### Measurements of ambulatory ECG ST-segment depression and covariates

We used the Burdick model 92513 Compact Digital Holter Recorder and Scanner/Software System (Burdick Inc., Deerfield, WI, USA). All technical specifications of ambulatory ECG monitoring followed recommendations of the American Heart Association (Knoebel 1989). Each morning, the subject removed leads and bathed before a research assistant arrived at the subject’s home. A well-trained research assistant downloaded ECG data and set up the ECG for a new daily run by reattaching the seven leads (V1 to V6 locations on the epigastrium and one reference electrode). Ambulatory ECG signals (three channels) were read and analyzed by Burdick Vision Premier Holter Analysis System, which includes algorithms for QRS labeling, artifact identification, and data correction. It also includes identification of rate-related abnormalities and analysis of ST changes. The ambulatory ECG data were edited by a well-trained technician from the University of California–Irvine Noninvasive Laboratory. A cardiologist then over-read and flagged ECG regions with indications of abnormalities, arrhythmias, and ST-segment changes.

For analyses of ECG ST-segment changes, only beats classified as normal and not preceded by ectopic beats or prolonged RR intervals were included. Reference lines were calculated by the Burdick software from the isoelectric line and 24-hr median of the ST-trend curve, as previously described (Quintana et al. 1995). Software identified ST-segment depression of ischemic type to be a horizontal or down-sloping shift of ≥ 1.0 mm (0.1 mV), occurring 80 msec after the J point and lasting > 60 sec. We counted episodes as unique if separated from previous episodes by at least 5 min.

We simultaneously monitored physical activity with an actigraph (Mini-motionlogger; Ambulatory Monitoring, Inc., Ardsley, NY, USA), which was placed near the waist. It records three axes of movement with high test–retest reliability and validity (Patterson et al. 1993). Actigraph data (1-min epochs) were normalized by subject-specific *z*-scores and then averaged across the current and 1 lagged hr for each hourly ST-segment depression measurement. Subjects also answered questions in the diary, including anginal symptoms, their physical location each hour (at home in the retirement community or elsewhere), and moderate to strenuous physical exertion. However, these questions were answered only during waking hours, in contrast to the ambulatory ECG and actigraph, which were worn for most of each day.

### Air pollutant exposures

Measurement methods are detailed elsewhere (Arhami et al. 2006, 2009; Delfino et al. 2009; Polidori et al. 2007, 2009). We conducted outdoor air monitoring at each retirement community as an approach to enhance the accuracy of exposure assessments compared with ambient air data. This also afforded us the opportunity to better characterize PM exposures. We measured hourly total PM_2.5_ OC and elemental carbon (EC) using the OC_EC Analyzer (model 3F; Sunset Laboratory Inc., Tigard, OR, USA) and then estimated exposure to carbonaceous aerosol classes characterized by primary and secondary sources using the “EC tracer method” (Cabada et al 2004; Polidori et al. 2007). Primary organic aerosol was estimated by primary OC assuming that primary OC and EC are emitted from the same combustion sources, which are mostly vehicular traffic in the Los Angeles study region. Periods dominated by primary sources were identified as hours with high levels of CO and NO that were primarily observed during morning rush hour traffic, when secondary OC is less likely to be formed. By regressing the OC and EC data collected during these periods, the characteristic primary OC:EC ratio for each month of study was determined. Primary and secondary OC was estimated by the following expressions:


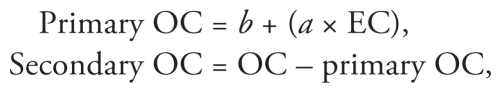


where *a* is the characteristic primary OC/EC ratio for the study area, and *b* is the intercept from the regression (assumed to be noncombustion primary OC).

We also measured hourly PM_2.5_ black carbon (BC) by aethalometer (Magee Scientific, Berkeley, CA, USA) as another indicator of primary carbonaceous aerosols. Given the strong similarity of BC with EC, we report results for BC only.

Other hourly PM measurements included total particle number (Condensation Particle Counter model 3785; TSI Inc., Shoreview, MN, USA), and PM_2.5_ mass (Beta-Attenuation Mass Monitor, model 1020; Met One Instruments Inc., Grants Pass, OR, USA). Pollutant gases (O_3_, NO_x_, and CO) were measured hourly using federal reference methods.

We measured daily size-fractionated PM mass concentrations with the Sioutas Personal Cascade Impactor Sampler (SKC, Inc., Eighty Four, PA, USA), including particles 0–0.25 μm in diameter (PM_0.25_), accumulation mode particles 0.25–2.5 μm in diameter (PM_0.25–2.5_), and coarse mode particles 2.5–10 μm in diameter (PM_2.5–10_) (Misra et al. 2002). We refer to PM_0.25_ as “quasi-ultrafine” because the cut-point for the ultrafine mode is usually considered around 0.1–0.2 μm.

### Statistical analysis

We used generalized estimating equations (GEEs) to model the discrete correlated response data of interest (Zeger and Liang 1986). GEE logistic models were used to estimate the odds of hourly ST-segment depression ≥ 1.0 mm from hourly to cumulative hourly air pollution exposures. Log-linear GEE models using a Poisson mean–variance relationship were used to estimate the relation of daily counts of ST-segment depression ≥ 1.0 mm to daily average air pollution exposures. We estimated regression parameters using the general linear model procedure in SAS (version 9.3; SAS Institute Inc., Cary, NC, USA). We assessed the fit of various GEE models with the quasi-likelihood under the independence model criterion (QIC) statistic (Pan 2001). For all models, we controlled for temperature, day of week, time of day (1100 to 1700 hours vs. 0600 to 2000 hours, community, and 6-week season. We decided *a priori* to control for temperature at the same averaging time as the air pollutant. We used smoothed penalized spline terms to adjust for nonlinear effects of temperature (Zanobetti et al. 2009b). Model fit by QIC was optimal for temperature splines with 3 or 4 knots. We found no confounding by medications, sex, or myocardial infarction history because the change in coefficients after adding these variables was < 10%. For the hourly exposure–response models, we additionally controlled for physical activity using actigraph data for the current hour. GEE models for time-varying predictors (air pollutants) were best fit with an autoregressive-1 working correlation matrix; however, final inference used the sandwich estimator to guard against misspecification of the assumed covariance structure.

For exposure variables, although we collected 24-hr filter-based gravimetric PM mass measurements, they did not match the hourly time frame of the ambulatory ECG monitoring. Therefore, we used only hourly air pollutant measurements in the analysis of risk of ST depression in each hour. We assessed more acute versus cumulative exposure–response relationships by testing the last 1-hr, 8-hr, and 24-hr (1-day) moving averages as well as cumulative exposures up to 9 days before the hourly ECG measurements. For the analysis of daily size-fractionated PM mass data, we assessed the rate ratio of ST-segment depression counts during the 24-hr monitoring period. The daily counts were related to the current day’s and previous day’s 24-hr average PM mass, but additional lags were not available because monitoring began the first day of each 5-day ECG session.

Physical exertion is associated with increased risk of cardiac ischemia in susceptible individuals and is expected to increase the dose of air pollutants. Therefore, it could modify the association between air pollution exposure and cardiac ischemia. Therefore, we tested in product term models whether there were positive interactions between actigraph activity (current and lag 1 hr, subject-specific *z*-scores) and air pollution. As an alternate activity measure, we also tested interaction with subject mean-centered hourly heart rate. Finally, in exploratory analyses, we tested interactions of pollutants with medication use, sex, or history of myocardial infarction. Product terms with *p*-values < 0.1 and their consistency across lags and across similar exposures (e.g., markers of primary combustion) were taken to suggest possible effect modification. We also tested whether associations were strengthened when restricting analyses to times when the subject reported being home, and thus near air monitors. The subject was assumed to be home during nighttime hours when there were no diary reports.

We report results scaled to an interquartile range (25th to 75th percentile) increase in each air pollutant so that results can be compared between pollutants regardless of units of measurement or concentration range.

## Results

Table 1 lists subject characteristics. Subjects remained at home for 91% of the waking hours that they answered diaries. Subjects reported anginal symptoms only 12 times out of 4,216 hourly diary entries. Two of those reports showed ST-segment depression ≥ 1.0 mm during the current or previous hour. We observed no difference in the frequency of ST depression by diary-reported moderate to strenuous activity.

Table 2 gives descriptive statistics on air pollution concentrations, which for the U.S. Environmental Protection Agency criterion air pollutants (PM_2.5_, NO_2_, CO, and O_3_) are fairly typical of most large urban areas (U.S. Environmental Protection Agency 2011). Markers of primary combustion (primary OC, BC, CO, and NO_x_) were strongly correlated with each other and moderately correlated with quasi-ultrafine PM mass (Table 3). O_3_ was inversely associated with these pollutants and showed a small positive correlation with secondary OC, likely because they both originate from photochemical processes (Polidori et al. 2007).

**Figure 1:**
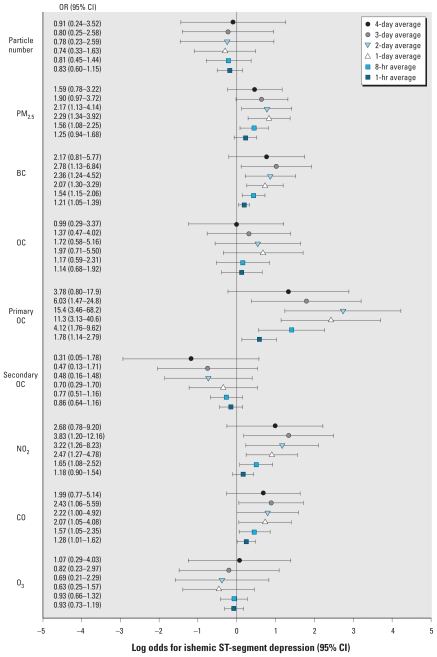
Odds ratios (ORs) of ST-segment depression ≥ 1.0 mm from exposure to hourly outdoor community air pollutants. Log odds and 95% CIs are for interquartile range increases in the air pollutant (Table 2), adjusted for average actigraph-derived physical activity for the same hour as the ST-segment depression outcome, temperature averaged over the same time as the pollutant, day of week, seasonal study phase, and community group.

In GEE regression analyses, we found positive associations of hourly ST-segment depression events with interquartile range increases in markers of primary combustion aerosols (BC and primary OC) and combustion-related gases (CO and NO_2_/NO_x_) [Figure 1; see also Supplemental Material, Table 1 (doi:10.1289/ehp.1002372)]. These associations were statistically significant (*p* < 0.05) for nearly all models for these pollutants averaged over the last 1 hr through 3–4 days. Estimates of association dropped toward 1.0 or confidence limits widened considerably at 7- to 9-day moving averages (not shown). The strongest association was for primary OC, with odds ratios as high as 15.4 for 2-day averages [95% confidence interval (CI), 3.5–68.2]. NO_2_ was more strongly associated with ST depression than was NO_x_. PM_2.5_ was positively associated with hourly ST depression for 8-hr through 3-day averages.

**Figure 2:**
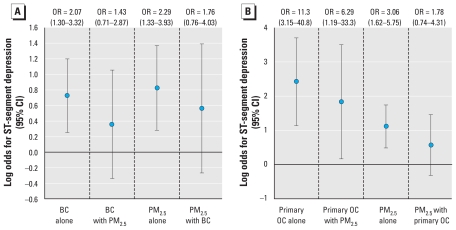
One- and two-pollutant regression models for the association of hourly ST-segment depression ≥ 1.0 mm with a marker of primary carbonaceous aerosols versus PM_2.5_: 24-hr average BC and PM_2.5_ (*A*) and 24-hr average primary OC and PM_2.5_ (*B*). Odds ratios (ORs) and 95% CIs are for interquartile range increases in the air pollutant (Table 2), adjusted for the same variables as in Figure 1, and for observations where the other pollutant is nonmissing (this resulted in slight differences from the estimates shown in Figure 1).

To assess whether the association with PM_2.5_ mass was explained by primary combustion aerosols, we coregressed PM_2.5_ with BC and with primary OC (Figure 2). The association of PM_2.5_ with ST depression was confounded by BC (32% decrease in log odds for PM_2.5_) and by primary OC (48% decrease). We observed a smaller decrease in the estimated regression coefficients for primary OC (24%), which remained significant, but regression coefficients for BC decreased more than PM_2.5_ (51%).

We observed largely consistent associations for the GEE Poisson models of the relation between the daily counts of ST-segment depression and daily average air pollutants [see Supplemental Material, Table 1 (doi:10.1289/ehp.1002372)]. In addition, the relative rate of ST-segment depression was significantly positive for 2-day averages of quasi-ultrafine (PM_0.25_) mass [rate ratio (RR) = 1.57; 95% CI, 1.19–2.06] and borderline significant for 1-day averages (RR = 1.17; 95% CI, 0.99–1.40), but not for larger particle size fractions (2-day average PM_0.25–2.5_: RR = 1.17; 95% CI, 0.70–1.95; 2-day average PM_2.5–10_: RR = 1.25; 95% CI, 0.94–1.67) (see Supplemental Material, Table 1 and Figure 1 (doi:10.1289/ehp.1002372)].

When we restricted models to the 91% of daytime hours that subjects reported being home (plus all remaining nighttime hours), associations were nearly unchanged. We observed no significant or consistent evidence of effect modification of associations between ST depression and primary air pollutant exposures by actigraph-measured physical activity, heart rate, sex, history of myocardial infarction, or medication use (statins, β-adrenergic receptor blockers, calcium channel blockers, angiotensin I-converting enzyme inhibitors/angiotensin II receptor antagonists) (data not shown).

## Discussion

This is the first study to our knowledge using 24-hr ambulatory ECG data that reports evidence of associations between ≥ 1.0 mm ST-segment depression (likely indicative of cardiac ischemia) and traffic-related air pollutant exposures. We found positive associations of ST-segment depression ≥ 1.0 mm with outdoor home exposures to markers of primary combustion aerosols and gases, but not with secondary OC or O_3_. This is also the first study to our knowledge to demonstrate that quasi-ultrafine PM, but not larger PM, is associated with ambulatory ST-segment depression. These observations are coherent with our previous findings of associations of weekly measurements of systemic biomarkers of inflammation (e.g., interleukin-6) with quasi-ultrafine PM and markers of primary organic aerosols (Delfino et al. 2008, 2009), including polycyclic aromatic hydrocarbons (PAHs) (Delfino et al. 2010b).

Several mechanisms may account for our findings of both very acute associations from exposure in the previous 1–8 hr and stronger associations using multiday averaging times. Short-term effects are supported by experimental data by Mills et al. (2007) showing that diesel exhaust exposure immediately enhances exercise-induced ST-segment depression in subjects with prior myocardial infarction. Those authors hypothesized that diesel-exhaust inhalation leads to oxidative stress and a subsequent reduction in nitric oxide availability, resulting in microvascular dysfunction in the resistance vessels of the myocardium. Short-term effects also may be mediated by autonomic nervous system responses via effects of pollutants on airway receptors (Brook et al. 2009), which is plausible because sympathetic activation can produce coronary vasoconstriction (Martin et al. 1989). In another experimental study, impairment of vascular function after brief controlled exposure to diesel exhaust appears to persist for up to a day (Törnqvist et al. 2007). Sustained high exposures to ambient PM over many hours or days may promote oxidative stress and inflammation leading to sensitization of coronary arteries to endogenous vasoconstrictors (Sun et al. 2008), potentially including endothelin‐1 (Langrish et al. 2009). In the present study, associations of ST depression with markers of primary organic aerosols and related gases were progressively stronger for longer averaging times going from 1 hr to several days. This suggests that much of the underlying ischemic effect could result from a cumulative progression of vascular inflammatory changes and perhaps endothelial dysfunction. This differs fundamentally from the very acute effect of exercise on ischemia, which was not apparent in the data. This inference is coherent with our previous findings of associations with biomarkers of inflammation that we observed with multiday averages of primary OC but not recent hourly exposures (Delfino et al. 2008, 2009). We also previously observed associations of ambulatory systolic and diastolic blood pressure with BC and primary OC that were substantially stronger for multiday moving averages than hourly or 24-hr exposures (Delfino et al. 2010c).

We found no association between ST-segment depression and particle number concentration, which is presumed to be dominated by nanoparticles. Although this finding is inconsistent with our earlier finding of positive associations between systemic biomarkers of inflammation and particle number (Delfino et al. 2008, 2009), it is consistent with our previous observation of no overall association between blood pressure and particle number (Delfino et al. 2010c). This lack of association may occur because quasi-ultrafine PM mass is dominated by the larger size fraction of that mode that has lower number concentrations than nanoparticles, which contribute little to mass but dominate particle numbers. We speculate that the chemical composition across this mass fraction is more important than number concentration or mass. We previously reported that blood biomarkers of systemic inflammation measured weekly were associated with PM_0.25_ PAHs from weekly particle filter composites, including all major PAH molecular-weight size fractions (Delfino et al. 2010b). Interestingly, when we coregressed total PAH with PM_0.25_ mass, total PAH completely confounded associations between biomarkers of inflammation and mass, but mass did not confound PAH, suggesting that PM chemical composition is important (Delfino et al. 2010b).

CO was positively associated with ST-segment depression. The observed low ambient concentrations of CO (≤ 1.68 ppm) are unlikely to have resulted in a direct effect on cardiac ischemia because magnitudes higher exposures to CO (leading to 2–3.9% carboxyhemoglobin) are required to induce a reduction in the time to ischemic ST-segment changes and anginal symptoms during exercise in subjects with coronary artery disease (Allred et al. 1991). Therefore, CO likely served as a surrogate for other causal pollutant components from primary combustion sources. Similarly, NO_2_ likely functioned as a surrogate indicator because NO_2_ by itself does not impair vasomotor function in humans (Langrish et al. 2010).

Our results are consistent with findings in other panel studies using nonambulatory (in-clinic) ECG data that measured ambient PM_2.5_ mass (Gold et al. 2005; Lanki et al. 2008; Pekkanen et al. 2002) and personal PM_2.5_ (Lanki et al. 2008). We provide new data suggesting that the effect of PM_2.5_ is more clearly linked to combustion-related PM, particularly to unregulated PM < 0.25 μm. The accumulation mode fraction of PM_2.5_ (PM_0.25–2.5_), composed of secondary particles, was not associated with ST-segment depression.

In the only other panel study employing ambulatory ECG monitoring, investigators found that an interquartile increase in the previous 24-hr mean ambient BC (0.47 μg/m^3^) was associated with a higher risk (1.50) of ECG-measured ST-segment depression ≥ 0.1 mm (RR = 1.50; 95% CI, 1.19–1.89) in 48 subjects with coronary artery disease living in Boston, Massachusetts (128 daily ambulatory ECGs) (Chuang et al. 2008). The authors showed somewhat weaker associations of ST-segment depression ≥ 0.1 mm with an interquartile increase in 1-day average PM_2.5_ (6.9 μg/m^3^; RR = 1.22; 95% CI, 0.99–1.50). This last difference in BC and PM_2.5_ is consistent with what we observed, but our results are not directly comparable because the Boston study used a continuous scale to detect ST changes. The present study and that of Chuang et al. (2008) are likely reporting the same phenomenon. However, Chuang et al. (2008) reported on risk of ST depression of ≥ 0.1 mm, which is less likely to represent ischemic changes than our findings for ST depression scaled at ≥ 1.0 mm (more typically used to define cardiac ischemia). The present study involved more data (328 daily ambulatory ECGs in 38 subjects) and exposures near the subjects’ residences, which may have allowed us to observe pollutant associations with ST-segment depression ≥ 1.0 mm. Another analysis of the Boston cohort panel showed positive associations of T-wave alternans (a marker of cardiac electrical instability) from the ambulatory ECGs with exposure to 2-hr average indoor BC (when at home), 2-hr ambient BC (when away), and being in traffic (Zanobetti et al. 2009b).

The lack of positive associations of ST-segment depression with O_3_ in the present study is consistent with a review of findings from time-series studies of daily hospital data for myocardial infarction in which 3 of 12 studies reported unexpected protective effects of ambient O_3_, and only two reported positive associations with O_3_ (Bhaskaran et al. 2009). The biologically implausible finding of an O_3_ protective effect is likely related to the inverse correlation often found between O_3_ and exposure markers of primary combustion.

We found no other epidemiologic studies with specific information to compare with our lack of apparent adverse effects from secondary OC, which is a marker of secondary (photochemically aged) organic aerosols. This is surprising in that photochemical transformations of aged primary combustion emissions enhance the redox activity of PM (Verma et al. 2009). However, we have presented evidence that NO_x_ and chemical components related to the primary combustion of fossil fuel have a greater impact on systemic inflammation, whereas O_3_ and chemical components related to secondary photochemical aging of PM have a greater impact on airway inflammation (Delfino et al. 2010a). In addition, we have reported that ambulatory systolic and diastolic blood pressure in the present panel of subjects was more weakly associated with secondary OC than with primary OC or BC and was not associated with O_3_ (Delfino et al. 2010c). This is consistent with experimental data in humans (Brook et al. 2009). Although both types of pollutants (combustion-related and photochemical) have oxidant potential, the target sites in the body may differ because of differing toxicokinetics such as the instantaneous reaction of O_3_ on airway surfaces and the water solubility of secondary organic chemicals (e.g., polar organics).

A limitation of the present study is the lack of hourly personal exposures. However, subjects generally remained near the home (91% of daytime hours) and thus near air monitors, and models restricting analyses to times at home were nearly unchanged. This can be compared with our study of ambulatory blood pressure that showed slightly stronger associations with analyses restricted to times at home (Delfino et al. 2010c). Furthermore, because indoor PM concentrations were strongly influenced by PM of outdoor origin, we focused only on outdoor data in the present analysis despite the fact that subjects spent most of their time indoors (86%). We previously reported that biomarkers of systemic inflammation were similarly associated with both outdoor and indoor PM_0.25_ PAHs (Delfino et al. 2010b), and similarly associated with both outdoor PM and estimated indoor PM of outdoor origin, including EC and primary OC (Delfino et al. 2008). Weekly averaged PM_0.25_ PAH and PM_2.5_ EC at the studied retirement communities showed indoor:outdoor ratios that were close to 1.0 and indoor–outdoor correlations that were strong (e.g., median *R* = 0.60 for 15 PAH species) (Arhami et al. 2010; Polidori et al. 2007). This is evidence that indoor PAH and EC were largely of outdoor origin and due to high penetration of these primary pollutants into indoor environments.

An unexpected finding was the lack of interaction with actigraph activity or heart rate, which may be surprising given that exercise testing typically is used to induce ST-segment depression and that exertion typically is associated with angina. However, there is evidence that mechanisms underlying ST depression under the conditions of exercise testing may differ from effects of other stresses in everyday life (Uen et al. 2008), which can be evaluated with ambulatory ECG data. Air pollutant–induced autonomic and inflammatory imbalances may be sufficient to induce ischemic responses. Another limitation is that we cannot be absolutely certain that the ST changes represent ischemia. However, we excluded patients with hypertrophy, digoxin use, bundle branch block, nonspecific ST–T wave abnormalities, and intraventricular conduction delay. Furthermore, there was no reason to expect acidosis, and certainly not repeated acidosis, particularly because no subject had uncontrolled diabetes, which would have led to recurrent patterns of ST-segment deviation. Therefore, although we cannot be certain that the ST-segment shifts of ≥ 1 mm truly represent ischemia, it is very likely that this was the underlying cause of the repolarization changes.

## Conclusions

Our results suggest that primary products of fossil fuel combustion lead to an increased risk of myocardial ischemia. This is consistent with the few time series studies that have found markers for these exposures such as ambient BC (Peters et al. 2001; Zanobetti and Schwartz 2006) as well as combustion-related gases (NO_2_/NO_x_ and CO) (Bhaskaran et al. 2009) are associated with daily hospital counts of myocardial infarction cases.

A key advantage of the present study is that we measured exposures in the outdoor home environment of subjects who remained near their homes throughout most of their follow-up. Furthermore, the continuous hourly measurements of both air pollutants and ST-segment depression using ambulatory ECG allowed us to explore the time span of exposure–response relations. Findings suggest very acute effects from exposure in the hours preceding the ST depression through several days of elevated exposures. Given the advanced age and history of coronary artery disease of our study subjects, we suspect that this may be a particularly susceptible population, but further study is needed in general populations to confirm this.

Our present and previous significant findings for both quasi-ultrafine PM and markers of traffic-related PM support the following hypothesis: Redox-active and other components in ultrafine PM from fossil fuel combustion directly or indirectly (via the lungs) affect cardiovascular target sites leading to systemic inflammation through oxidative stress and other mechanisms and thereby precipitate adverse cardiovascular responses, including increased blood pressure and cardiac ischemia in humans (Delfino et al. 2005).

## Figures and Tables

**Table 1:** Subject characteristics (*n* = 38).

Variable	*n* (%)
Sex	

Male	27 (71.0)
Female	11 (29.0)

Cardiovascular history	

Confirmation of coronary artery disease^a^	
Myocardial infarction	15 (39.5)
Coronary artery bypass graft or angioplasty	13 (34.2)
Positive angiogram or stress test	7 (18.4)
Clinical diagnosis^b^	3 (7.9)
Congestive heart failure	7 (18.4)
Hypertension	27 (71.0)
Hypercholesterolemia	31 (81.6)

Medications^c^	

β-Adrenergic receptor blockers	21 (55.2)
α-Adrenergic receptor blockers	4 (10.5)
Calcium channel blockers	12 (31.6)
ACE inhibitors and angiotensin II receptor antagonists	18 (47.3)
HMG-CoA reductase inhibitors (statins)	19 (50.0)

Abbreviations: ACE, angiotensin I-converting enzyme; HMG-CoA, 3-Hydroxy-3-methylglutaryl coenzyme A.

a Each category is hierarchical and excludes being in the above diagnostic category.

b Includes subjects with anginal symptoms relived with nitrates plus echocardiogram and ECG evidence of past infarct.

c Daily use of each prescription medication was reported by subjects in paper diaries daily. The subjects counted here took the medication on a regular daily schedule.

**Table 2:** Descriptive statistics of 24-hr average outdoor community air pollutant exposures.

Exposure (24-hr averages)	*n* (missing)	Mean ± SD	Interquartile range	Minimum/maximum
PM measurements^a^				

PM_2.5_ (μg/m^3^)^b^	235 (0)	21.1 ± 11.4	16.0	2.3/77.4
Particle number (no./cm^3^)	184 (51)	12,817 ± 5,889	6,351	2,019/30,180
BC (μg/m^3^)	235 (0)	1.67 ± 0.79	1.02	0.29/4.51
OC (μg/m^3^)	188 (47)	7.78 ± 3.68	5.20	2.46/18.7
Primary OC (μg/m^3^)	157 (78)	5.34 ± 2.92	4.37	1.41/12.5
Secondary OC (μg/m^3^)	157 (78)	2.90 ± 1.54	2.14	0.27/7.65

Size-fractionated PM mass (μg/m^3^)^b^				

PM_0.25_	217 (18)	9.77 ± 4.12	7.00	2.46/30.05
PM_0.25–2.5_	226 (9)	11.37 ± 9.40	10.58	0.98/66.77
PM_2.5–10_	217 (18)	9.38 ± 4.98	5.46	0.30/24.63

Outdoor hourly gases				

NO_2_ (ppb)	235 (0)	27.5 ± 11.6	17.4	2.9/58.1
NO_x_ (ppb)	235 (0)	46.6 ± 31.4	42.3	3.2/184
CO (ppm)	224 (11)	0.53 ± 0.30	0.42	0.01/1.68
O_3_ (ppb)	232 (3)	27.1 ± 11.5	17.4	3.8/60.7

a BC, PM_2.5_, and the gases had fewer missing observations because two samplers were operated in parallel at all times. Primary and secondary OC had more missing data than did total OC because of missing predictor data used to estimate these two OC fractions, including EC.

b PM_2.5_ mass was measured with a Beta-Attenuation Mass Monitor, whereas the size-fractionated PM mass was measured with a Personal Cascade Impactor Sampler, which had more missing data.

**Table 3:** Spearman correlation matrix for outdoor home air pollutant exposures.^a^

Pollutant	PM_2.5_	OC	BC	Primary OC	Secondary OC	PM_0.25_	PM_0.25–2.5_	PM_2.5–10_	NO_x_	CO	O_3_
Particle number	−0.13	0.27	0.40	0.47	−0.08	0.36	−0.12	0.06	0.63	0.45	−0.38
PM_2.5_	1.00	0.44	0.58	0.43	0.22	0.20	0.87	0.55	0.14	0.31	0.04
OC		1.00	0.63	0.65	0.72	0.41	0.33	0.33	0.46	0.59	−0.05
BC			1.00	0.88	0.07	0.52	0.43	0.44	0.83	0.79	−0.38
Primary OC				1.00	0.01	0.55	0.33	0.36	0.79	0.75	−0.36
Secondary OC					1.00	0.09	0.16	0.15	−0.09	0.11	0.26
PM_0.25_						1.00	0.17	0.35	0.51	0.54	0.01
PM_0.25–2.5_							1.00	0.60	0.01	0.13	0.08
PM_2.5–10_								1.00	0.18	0.26	0.06
NO_x_									1.00	0.82	−0.53
CO										1.00	−0.29

a All exposures are 24-hr averages and are mean-centered by retirement community and seasonal phase.
